# Harvesting Entropy for Random Number Generation for Internet of Things Constrained Devices Using On-Board Sensors

**DOI:** 10.3390/s151026838

**Published:** 2015-10-22

**Authors:** Marcin Piotr Pawlowski, Antonio Jara, Maciej Ogorzalek

**Affiliations:** 1 Institute of Information Systems, University of Applied Sciences Western Switzerland (HES-SO), Sierre 3960, Switzerland; E-Mail: jara@ieee.org; 2 Department of Information Technologies, Faculty of Physics, Astronomy and Applied Computer Science, Jagiellonian University, Krakow 30-348, Poland; E-Mail: maciej.ogorzalek@uj.edu.pl; 3 Research and Development Department, HOP Ubiquitous, 30562 Ceuti, Murcia, Spain

**Keywords:** random number generation, entropy, on-board sensors, Internet of Things

## Abstract

Entropy in computer security is associated with the unpredictability of a source of randomness. The random source with high entropy tends to achieve a uniform distribution of random values. Random number generators are one of the most important building blocks of cryptosystems. In constrained devices of the Internet of Things ecosystem, high entropy random number generators are hard to achieve due to hardware limitations. For the purpose of the random number generation in constrained devices, this work proposes a solution based on the least-significant bits concatenation entropy harvesting method. As a potential source of entropy, on-board integrated sensors (*i.e.*, temperature, humidity and two different light sensors) have been analyzed. Additionally, the costs (*i.e.*, time and memory consumption) of the presented approach have been measured. The results obtained from the proposed method with statistical fine tuning achieved a Shannon entropy of around 7.9 bits per byte of data for temperature and humidity sensors. The results showed that sensor-based random number generators are a valuable source of entropy with very small RAM and Flash memory requirements for constrained devices of the Internet of Things.

## 1. Introduction

In the near future, many people will benefit from emerging technologies, such as the Internet of Things (IoT) [1,2]. The IoT will consist of billions of highly-constrained devices with limited computing capabilities and interconnected by wireless communication mechanisms with remote systems [3]. A web of newly-connected machines will positively change our life standards [4,5], but at the same time, new security and privacy concerns will be introduced [6,7].

The research community has devoted a significant effort addressing the security issues of IoT networks [8]. The most notable efforts came from the Internet Engineering Task Force (IETF) Datagram Transport Layer Security for the Internet of Things (DTLS-IoT) [9] working group that has been addressing usage issues of the Transport Layer Security (TLS) protocol for the protection of end-to-end communication between constrained devices in IoT networks [10]. The Authentication and Authorization for Constrained Environments (ACE) IETF working group has been devoted to addressing the need of highly-secure and privacy-oriented standards for authorization and authentication in the IoT [11]. Other research community efforts have been addressing network layer security aspects of IoT networks [12,13,14,15] and also denial of service mitigation techniques [16].

The entropy of random number generators has been pivotal for the security of network communication [17]. Random values have been widely used in many places of the communication stack and operating systems, especially responsible for security mechanisms. Having an unpredictable source of random data might be the last line of defense against an adversary trying to crack applied security mechanism. High entropy random number generators could mitigate the threat of revealing sensitive information from a system. It is essential for the operation of security mechanisms to rely on a good source of randomness [18]. Different sources of randomness have been cryptographically-secured pseudo random number generators (PRNG) [19]. Such generators have been commonly used in many of the current security mechanisms (e.g., TLS, DTLS) and, in particular, during the key negotiation phase of the authentication mechanism (e.g., Extensible Authentication Protocol - Transport Layer Security (EAP-TLS), EAP - Pre-Shared Key (EAP-PSK) or EAP-Message Digest 5 (EAP-MD5)) [20,21,22]). Unfortunately, the PRNGs require being initiated with a special random value called a seed that determines the random sequence. The key to the security of the PRNG lies in the unpredictability of the seed. Generating such an unpredictable random value, in very constrained devices, has been a problematic task that has been addressed in this research.

This paper evaluates how much of the entropy could be harvested from the least significant bits of data returned by on-board sensory devices of constrained IoT devices. Finding a high entropy, fast and lightweight method for generating random data will lead to more secure communication and authentication mechanisms for constrained devices.

The humidity, temperature and two light sensors that have been incorporated into the TelosB-compatible [23] mote have been evaluated as potential sources of the entropy in conjunction with the proposed least-significant bits concatenation method for entropy harvesting. The presented approach has been tested in four different experiments and optimized using a statistical fine-tuning approach. Throughout the research, the Shannon and min-entropy estimators have been used to assess the amount of entropy. Although both estimators do not provide strong confidence in the real amount of entropy, they have been used with respect to the comparison with the results from related works. Additionally, time and memory requirements for the Contiki OS [24] of the proposed approaches have been measured.

The remainder of this paper is structured as follows: Section 2 introduces the definitions used throughout this publication. Section 3 presents the analysis of related works and the motivation behind the research. Section 4 has been devoted to the presentation of the proposed solution; it contains a description of the methodology used during the research, the presentation of the least-significant bits concatenation method for harvesting the entropy from sensor devices and, finally, theoretical justification of the idea behind the proposed method. Section 5 describes initial results, analysis and selection of the best method that has been used during the rest of the research, then the long-term entropy harvesting experiment results are presented. Afterwards, the isolated environment experiment results are presented, and at the end, the statistically fine-tuned approach is presented to show the improvement of the entropy results. Section 6 discusses differences between these results and previous research efforts. The paper ends with Section 7, which concludes the research and describes future work efforts.

## 2. Definitions

Random number generators or random bit generators can be divided into two categories, deterministic and non-deterministic. The definitions of random number generators, following notations from NIST recommendations [19,25], are presented below.

The deterministic random bit generator (DRBG) produces a pseudo-random sequence of bits that has been correlated with the initialization value. This value has been called the seed and, in its secrecy, the whole security of the DRBG lies. Two identical generators initiated with the same seed will produce identical outputs. Such DRBGs are very useful in communication security, but they will not be further analyzed in this research. Throughout this paper, the DRBG will be referred to as the pseudo random number generator (PRNG).

A second category of random number generators is non-deterministic random bit generators (NDRBG). NDRBG produce output with full (or at least very high) entropy. That means that the output sequence of the NDRBG is unpredictable. In this research, such random number generators have been pursued. Throughout this paper, the NDRBG will be referred to as random number generators (RNG).

The source of randomness has been evaluated by two entropy estimators, the best known Shannon entropy (Equation (1)) and NIST-recommended min-entropy (Equation (2)).

(1) $$H\left(X\right)=-\sum_{x}P\left[X=x\right]\log_{2}P\left[X=x\right]$$

(2) $$H_{\infty }\left(X\right)=-\log_{2}\left(\max_{x}P\left[X=x\right]\right)$$

where *X* is a discrete random variable; in the context of this paper, *X* is a string of eight bits. The particular eight-bit length of the random variable has been chosen from the perspective of the practical implementation of the random number generator for constrained devices. Additionally, extending the bit length of the *X* will also extend the time of entropy collection. In terms of random number generator applicability in the communication parts of the constrained operating system, the preference needs to be set to solutions that work more quickly and that have a smaller memory footprint. Extending the bit length of *X* would lead to a reduction of the functionality of the proposed solution. Furthermore, using *X* of a length of one byte during entropy estimations is important from the perspective of the comparison with related works. Relevant referenced works have used a one-byte resolution for entropy estimation; therefore, the same approach has been adopted in this research.

## 3. Related Works and Motivation

The work of Hennebert, Hossayni and Lauradox in [26] is the most relevant to this research. The authors have evaluated many on-board and external sensors (*i.e.*, temperature, humidity, magnetic, gas pressure, motion, vibration and acceleration sensors) as the potential source of the entropy. The evaluation of sensors has been executed in three modes of operation: stability, where the external conditions have not been changed, dynamic, where the external conditions have changed rapidly, and saturation, where the external conditions have been set to continuously extreme values. Additionally, the authors are the first to have used the NIST-recommended min-entropy to measure the amount of entropy.

The highest Shannon entropy measured in [26] has been obtained for the vibration sensor and has been estimated for 7.8 bits per byte of data. The measured min-entropy of the temperature sensors in [26] has been estimated as 3.55 pits per byte of data from the internal sensor and 4.8 bits per byte of data from the external sensor.

However, the authors have not measured the time required to gather a particular amount of entropy, and they also have not presented the results of the memory consumption of the proposed solutions.

In [27], Francillon and Castelluccia have shown that transmission bit errors on a wireless sensor network are a potentially good source of entropy. By designing and implementing a random number generator based on wireless transmission bit errors, the authors have shown that their approach is practical and feasible. They have measured and presented the time and memory requirements of the proposed solution.

ROM memory requirements for the solution presented in [27] have been measured as 11,086 bytes for the MICA2 platform and 9832 bytes for the MICAz platform. RAM memory requirements presented in [27] have been measured as 471 bytes for the MICA2 platform and 416 bytes for the MICAz platform. Processing times presented in [27] have been estimated at around 146.2 ms (with Electrically Erasable Programmable Read-Only Memory (EEPROM) operations) or 4.5 ms (without EEPROM operations), calculated as a sum of random number generation, initialization and entropy accumulation for getting one byte of entropy.

Unfortunately, the authors have not evaluated the amount of entropy for the presented random number generator.

Both related works have made interesting contributions to the problem of entropy harvesting in constrained environments. However, they have also missed complementary information (*i.e.*, time and memory measurements for [26] and entropy evaluation for [27]).

In [28], Fabbri and Callegari presented a very low-cost, robust, easy to deploy entropy source based on chaotic dynamics. The device is capable of generating at least 32 kbit/s of entropy and offers multiple serial communication options, including USB, I2C, SPI or USART. Its operation is based on a loop built around an analogue-to-digital converter hosted on a standard microcontroller. This solution has a very effective hardware-based approach, solving the problem of entropy gathering for constrained devices.

The contribution presented by Fabbri and Callegari is important, but since the solution is hardware based, it requires additional effort to be implemented in current IoT devices. The method presented in this paper is software based; therefore, it is much more flexible and easier to deploy than the hardware one.

In [29], Sunar, Martin and Stinson presented a provably-secure true random number generator with built-in tolerance to active attacks. The paper contributes to the theory of true random number generators based on sampling phase jitter in oscillator rings. They have shown that an exponential increase in the number of oscillators is required to obtain a constant factor improvement in the fill rate. The key idea of the paper has been the measurements of the fraction of the time domain in which the analogue output signal has been random.

The theoretical work by Sunar, Martin and Stinson is very interesting, stimulating and has allowed better conceptualization and theoretical analysis of the approach presented in this paper.

The presented related works addressed the problems of acquiring the entropy source and generating random numbers for constrained devices. The motivation behind this paper is similar, and it is focused on solving the problem of acquiring enough randomness for the initialization of pseudo random number generators for applications in security solutions for constrained IoT devices.

During the research on the authentication mechanisms for the IoT [20,21,22], one of the most important security-related functions is missing. The random number generator is failing. The same sequences of random values have come out from the *rand()* function that is provided by the Contiki operating system. The problem can be addressed by employing two different approaches.

The first approach is the design and application of the hardware-based RNG, which is probably the most effective way to provide a high entropy source for a system, but it requires physical modification of the devices. Additionally, it increases the power consumption, size and cost of a device.

The second approach is the design of the software solution. This kind of solution is a bit more challenging, because it is limited only to hardware resources that are already available on a device. The solution based on the software approach is more flexible and can be applied on already-designed devices, without physical intervention. Therefore, the already-deployed solution can benefit more easily from a software-based approach by a software update.

The software-based approach has been chosen as the most flexible and effortless way of providing an entropy source to the operating system of the constrained IoT devices. Looking at the TelosB [23] compatible devices, it has been noticed that the most promising source of entropy could be the on-board sensory devices. The sensory measurements are external to the system, therefore potentially unpredictable. For this purpose, available on-board sensor devices have been selected for the analysis of the amount of entropy that can be harvested from them. In this paper, on-board humidity, temperature and two light sensors have been selected and evaluated as the potential source of the entropy.

## 4. Proposed Solution

**Figure 1:**
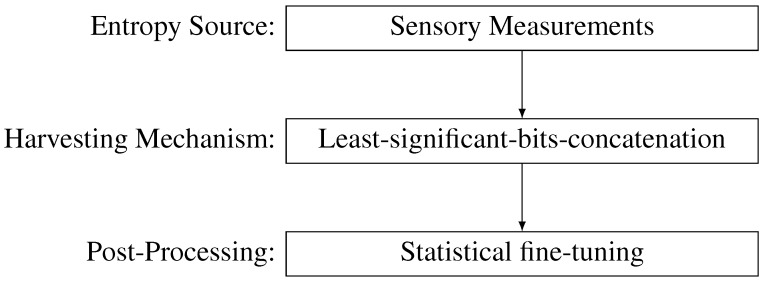
Simplified random number generation process with the particular building blocks proposed in this paper.

In this section, the proposed solution is presented. It begins with a description of the methodology used during this research, then the least-significant bits concatenation method is presented and, finally, theoretically analyzed. Figure 1 presents the simplified process of random number generation with the building blocks proposed in this paper.

### 4.1. Methodology

In this subsection, the methodology used during the research is presented, in which the experimental equipment and tools are described.

The platform used during this research is the TelosB-compatible [23] mote with the MSP430 MCU, 10 KB of RAM and 50 KB of ROM. All developed applications have been designed to work under the control of Contiki OS 3.x [24].

During this research, the on-board temperature, humidity, visible light and separate visible with infrared light sensors have been used. The technical details of the analyzed on-board sensory devices are presented in Table 1.

**Table 1:** Technical specifications of the analyzed on-board sensors.

Sensor	Part	Properties
Light (1)	Hamamatsu S1087 series	Visible range (560-nm peak sensitivity wavelength)
Light (2)	Hamamatsu S1087 series	Visible and infra-red range (960-nm peak sensitivity wavelength)
Temperature	Sensirion SHT11	Range: −40∼123.8 °C
		Resolution: ±0.01 (typical)
		Accuracy: ±0.4 °C (typical)
Humidity	Sensirion SHT11	Range: 0%∼100% RH
		Resolution: 0.05 (typical)
		Accuracy: ±3% RH (typical)

Additionally, the *entropy* [30] software packet for the R programming language and software environment for statistical computing and graphics [31] has been used for the calculation of both entropy estimators and also to process and graphically represent the collected data.

The presented approach is theoretically analyzed in Section 4.3 and extensively evaluated during the experimental analysis in Section 5.

### 4.2. Least-Significant Bits Concatenation Method

The entropy harvesting methods proposed in this paper have been designed to determine the amount of entropy that could be provided by concatenating the least-significant bits of the data returned by the analyzed on-board sensory device.

Four entropy-harvesting methods have been designed for this research. All of them are based on the observation of the instability (unpredictability) of the least-significant bits of the returned data from the on-board sensor devices. This instability has been related to various factors, such as analog to digital converter precision, electronic noise, measurement accuracy and external environmental influences, like small temperature or light variations that, by their nature, are unpredictable.

The most straightforward approach exploiting that observation is represented by the following Algorithm 1:
**Algorithm 1** Entropy harvesting mechanism for particular method and sensor type.**Input:**
*M* is the number of the method, *T* is the type of the sensor.**Output:**
*e* is one byte of the entropy. *e* ← 0 *N* ← ⌊8 ÷ *M*⌋ **for**
*i*
**from** 0 **by** 1 **to**
*N* − 1 **do**
 *s* ← *read_sensor*(*T*)*t* ← *least_significant_bits*(*s*,*M*)*e* ← *e* || *binary_shift_left*(*t*, (*i* ∗ *M*) **end for** **if**
*M* == 3 **then**
 *s* ← *read_sensor*(*T*)*t* ← *least_significant_bits*(*s*,2)*e* ← *e* || *binary_shift_left*(*t*,6) **end if**


Where *M* is the number of the method, *T* is the type of the sensor, *read_sensor*(*T*) returns the output data from a particular sensor, *least_significant_bits*(*a*, *b*) returns *b* least-significant bits from the input *a* and *binary_shift_left*(*a*, *b*) returns a value that is equal to value *a* left-shifted by *b* bits.

The first method (*M* = 1) obtains only one least-significant bit of every datum returned from the sensor device and concatenates gathered bits until they form an output byte: the sensor needs to be read eight times to build one byte of random data. The second method (*M* = 2) takes two least-significant bits from every value returned from sensor device, to build the one random byte it requires to read the sensor device four times. The third method (*M* = 3) reads the sensor data twice and takes three least-significant bits from every sensor value returned, then reads the sensor one last time and takes two least-significant bits; this requires accessing the sensor three times to generate one byte of entropy. The fourth method (*M* = 4) takes four least-significant bits from every sensor datum request and builds the output random byte; this requires reading the sensor data twice to build one random byte.

### 4.3. Theoretical Analysis

In Section 4.2, the least-significant bit concatenation method of gathering entropy from on-board sensor devices is presented. This section is devoted to the theoretical justification of the proposed method.

**Figure 2:**
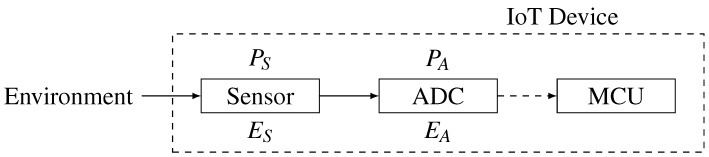
The simplified sensory measurement communication path. The *P_S_* and *P_A_* represent the precision of the sensor and ADC devices, respectively. The *E_S_* and *E_A_* represent the error measurement factor of the sensor and ADC devices, respectively. The straight line represents the analogue signal, and the dashed line represents the digital signal.

A simplified environment measurement path, shown in Figure 2, has been designed to represent the process of gathering information from the on-board sensory device. The path begins from the environment interfaced with the sensor; the sensor measures the environment and sends the result to the analogue-to-digital converter (ADC), which finally presents a digitized measurement to the microcontroller of the IoT device.

The sensor and the ADC nodes on the measurement path are points of interest in terms of the introduction of entropy into the path. The sensor device consists of an input that is responsible for taking measurements of environmental conditions. The input from the environment by its nature is an analogue signal with unlimited precision that cannot be precisely measured by any sensory device. In addition, the environment signal is spatially distributed, and its value varies depending on the place and the time of the measurement. The perception of an environmental condition highly depends on the precision of the measurement. The sensory device has an internal measurement precision (*P_S_*) that represents the quality of the sampled environmental information. The measured information by the sensory device, due to the internal measurement precision, introduces the sensor device measurement error (*E_S_*), which is also related to many additional factors, such as electronic noise or natural physical deviations of the sensory device that are unpredictable and of a chaotic nature. The sensor device measurement error is the first internal candidate that introduces the chaotic factor to the measurement path. The ADC device takes an analogue input from the sensor and converts it into the digital domain. It has its own internal precision (*P_A_*) that dictates the quality of the measurement. By its nature, such a conversion is bound to lose precision due to the change of the representation of the measured information from the analogue to the digital domain. This introduces measurement errors (*E_A_*) coming from the conversion itself and an internal electronic noise or natural physical deviations of the ADC device that are unpredictable.

Assume that *P_S_* and *P_A_* represent the bit length of the measured information for the sensory and ADC devices, respectively, and *E_S_* and *E_A_* represent the error measurement factor; then, $\frac{P_{S}}{E_{S}}$ and $\frac{P_{A}}{E_{A}}$ represent the length of the error-less substring of the measured environmental information. This infers that error measurement factors cannot be equal to zero; then, $P_{S}>\frac{P_{S}}{E_{S}}$ and $P_{A}>\frac{P_{A}}{E_{A}}$, which means that at least one bit of the bit-string has been corrupted, hence coming from a chaotic source, and it can be utilized as the source of entropy.

In the general case, the length of the substring of errors coming from the ADC might differ from $P_{A}-\frac{P_{A}}{E_{A}}$, since the ADC has taken as an input the sensory output. The only case when the sensory device error does not propagate is the situation in which the error-less substring of the sensory device is longer than the precision of the ADC ($\frac{P_{S}}{E_{S}}>P_{A}$). In that case, the error will be introduced only by the ADC conversion, which still should be suitable as the source of entropy.

It should be mentioned that the least-significant bits concatenation method presented and analyzed above has not been designed as an equivalent of a complete random number generator, but only as a way of collecting more entropy from the sensory device. Additionally, the amount of entropy can be increased by employing post-processing methods, such as the von Neumann corrector [32], XOR-tree or SHA-1 [32].

Two factors impact the amount of entropy gathered using least-significant bits concatenation, the type of environment measurement and the sensory device (including the ADC). The value presented to the microcontroller is the superposition of both factors. Assume that either the environment or the sensory device does not introduce any errors. If the environment gives no entropy, then the sensory device would introduce deviations to the measurement, hence creating chaotic information. If the sensory devices were perfect and did not introduce any errors into the measurements, then the environment would introduce spatial and time variations of a chaotic nature.

Therefore, the assumption that there has been an accumulation of the chaotic bits in the least-significant parts of the bit string is theoretically justified, and it is experimentally substantiated in the next section.

#### Side Channels Attacks

For the purpose of this analysis, side channel attacks have been divided into two categories, internally oriented and externally oriented.

The internally-oriented attacks focus on all aspects of information coming from the attacked device, such as timings [33], power [34], electromagnetic [35] or even sound side channels [36]. Employing such a side channel attack is a powerful way of breaking the security mechanism of the attacked device, rendering even the most powerful cryptographic algorithms useless [36]. Due to limited scope of this paper, internally-oriented side channel attacks have not been analyzed any further, but it should be noted that the presented solution does not employ any countermeasures against these attacks and is potentially vulnerable to all of them.

The second category is externally-oriented side channel attacks, which are attacks on the device sensors, introducing environmental changes to disturb and eventually guess the outcome of the random sequence harvested from the sensory device. This type of attack can be employed on the constrained device under the condition that most of the entropy coming from the sensory device is strongly correlated with an environment and weakly correlated with internal noises. In such a case, where only the environment dictates the outcome of the random sequence, the possibility exists to manipulate the environment in such a manner that the attacker could obtain the correlated random number sequence from more than two devices. The first attempt to achieve such a result would be to put the attacker device in very close proximity to the target device, to make the differences in the environment as few as possible. With the assumption that the sensory device is perfect, which means that it is not introducing any internal noises, the outcome of such a manipulation could lead to correlated entropy sequences. Another attempt could be employed by environmental parameter manipulation, which could lead to uncovering correlations in the random sequences that have not been observed during the regular operation of devices.

All side channel attacks should be considered as applicable attacks on constrained devices. The presented entropy-harvesting solution has not been designed to prevent any of these security threats, but it has been designed to solve a particular problem of gathering entropy for random number generation using constrained devices without hardware modifications. Therefore, the prevention mechanisms for side channel attacks on the presented method of entropy harvesting has been left for further research.

## 5. Experimental Analysis

This section is devoted to the performance analysis of the designed methods. At first, the best method in terms of the highest entropy results is selected. Then, the selected method is validated during a long-term experiment. At the end of this section, the selected method is analyzed in an isolated environment.

The following experiments have been designed to determine which of the defined methods give the highest entropy values. This is achieved by evaluating the Shannon entropy and min-entropy of the gathered random values. Additionally, the time required to collect one byte of entropy is measured and presented. RAM and Flash memory usage is calculated and presented to show how much of the resources are needed, in particular the conjunction of the methods with the sensory devices.

All experiments are performed in stable conditions, a home-office environment, without introducing any extreme factors during the measurements. The simulation environment is devised in such a manner that it reflects the conditions of large quantities of IoT devices deployed in natural small-office/home-office environments.

### 5.1. Best Method Selection

During this experiment, the proposed methods are evaluated, and the best method in terms of the highest entropy results is selected. In this subsection, initial experimental results are presented and analyzed. At the beginning, the time results are discussed, then memory consumption results are presented, and at the end of the section, the entropy measurement results are analyzed.

The first experiment is designed to determine the time required to collect one byte of entropy from the analyzed sensors. It is noted that the longest entropy collecting time is measured for the temperature sensor. The temperature sensor based first on the harvesting method takes almost 1.7 s to collect one byte of entropy. The second longest entropy gathering time is observed for the humidity sensor, and it is around 0.5 s. Both sensors are based on the same on-board electronics, Sensirion SHT11, which is the main factor of the delay.

For the temperature and humidity sensors, the entropy collection time for the fourth method is reduced by about four times. Clearly, the main factor contributing to the time reduction is the fact of the decrease of the sensory data reads.

The fastest entropy collecting time is recorded for the light sensors, and they are around 1.2 ms when the first harvesting method was used. By applying the fourth entropy-collecting method, the random byte harvesting time is reduced by 50%. The similar time results for the light sensors are related to the fact that both use the same electronics, Hamamatsu S1087 Series.

The full processing time comparison of different entropy-harvesting methods is presented in Table 2.

**Table 2:** Time required to collect one byte of entropy from the analyzed sensors with particular harvesting methods.

Sensor	Method 1	Method 2	Method 3	Method 4
Temperature	1698 ms	850 ms	636 ms	424 ms
Humidity	512 ms	253 ms	188 ms	127 ms
Light (1)	1.19 ms	0.85 ms	0.70 ms	0.67 ms
Light (2)	1.22 ms	0.85 ms	0.76 ms	0.70 ms

The second experiment is designed to measure the memory consumption of the entropy-gathering function in comparison to the sensory device and method in use. Similar to the time experimental results, a difference is observed between Sensirion SHT11- and Hamamatsu S1087 Series-based sensory devices. The entropy-harvesting methods using SHT11-based sensory devices (which are the humidity and temperature sensors) have flash memory requirements of around one kilobyte and RAM requirements of just one byte. The entropy-gathering methods that use the S1087 series-based devices (light sensors) have 50% lower flash memory requirements of around 500 bytes, but have higher RAM usage of four bytes.

The full comparison of the Flash and RAM memory consumption of the different entropy-harvesting methods is presented in Table 3.

**Table 3:** Flash and RAM memory requirements for gathering entropy with particular harvesting methods.

Sensor	RAM	Flash			
		Method 1	Method 2	Method 3	Method 4
Temperature	1	966	988	998	976
Humidity	1	966	988	998	976
Light (1)	4	488	510	520	498
Light (2)	4	488	510	520	498

The last experiment is devoted to estimating the values of the Shannon entropy and min-entropy of the gathered random bytes.

The highest Shannon entropy is observed for Method 1. The min-entropy has also the highest values for the first method, with the exception of the second method for the Light (2) sensor (this might be related to the insufficient number of samples). The differences between the values of estimated entropies and other methods are less significant. An interesting observation is made while analyzing the entropy of the data collected from the light sensors. There is a very prominent differentiation between the first two methods and the last two methods for both the min-entropy and the Shannon entropy.

The highest Shannon entropy is observed for the temperature sensor. The min-entropy is high for the temperature sensor, but also for both light sensors, with the exception of the third and fourth method. An interesting observation is made for the results of the random data from the humidity sensor, where the differences between the Shannon entropy and the min-entropy are the most significant.

**Figure 3:**
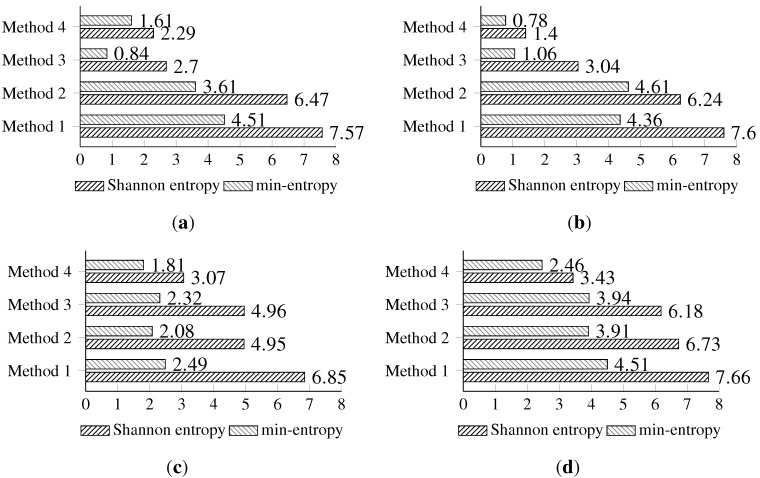
The comparison of the measured Shannon and min-entropy of the initial experimental results. (**a**) Light (1); (**b**) Light (2); (**c**) humidity; (**d**) temperature.

**Figure 4:**
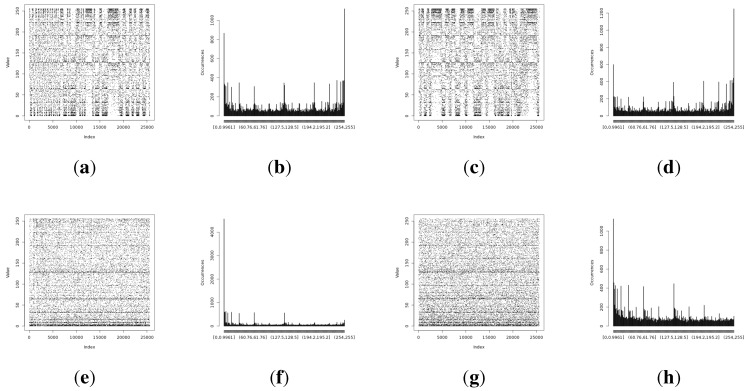
Initial experimental results of the harvested entropy by the first method in conjunction with the particular on-board sensor. (**a**) Light (1) sensor entropy; (**b**) distribution from the Light (1) sensor; (**c**) Light (2) sensor entropy; (**d**) distribution from the Light (2) sensor; (**e**) humidity sensor entropy; (**f**) distribution from the humidity sensor; (**g**) temperature sensor entropy; (**h**) distribution from the temperature sensor.

**Figure 5:**
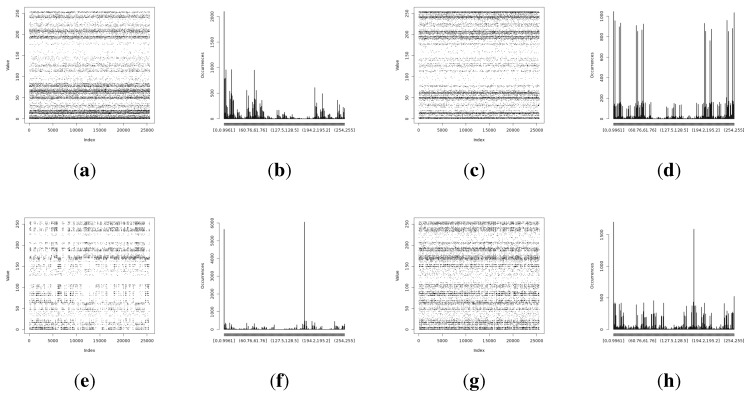
Initial experimental results of the harvested entropy by the second method in conjunction with the particular on-board sensor. (**a**) Light (1) sensor entropy; (**b**) distribution from the Light (1) sensor; (**c**) the Light (2) sensor entropy; (**d**) distribution from the Light (2) sensor; (**e**) humidity sensor entropy; (**f**) distribution from the humidity sensor; (**g**) temperature sensor entropy; (**h**) the distribution from the temperature sensor.

**Figure 6:**
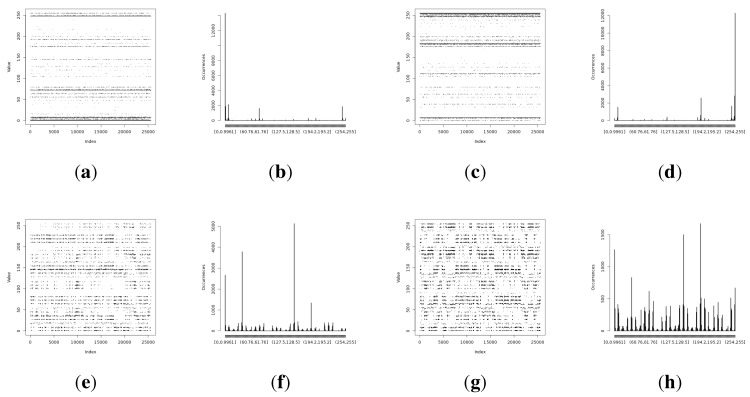
Initial experimental results of the harvested entropy by the third method in conjunction with the particular on-board sensor. (**a**) Light (1) sensor entropy; (**b**) distribution from the Light (1) sensor; (**c**) the Light (2) sensor entropy; (**d**) distribution from the Light (2) sensor; (**e**) humidity sensor entropy; (**f**) distribution from the humidity sensor; (**g**) temperature sensor entropy; (**h**) the distribution from the temperature sensor.

**Figure 7:**
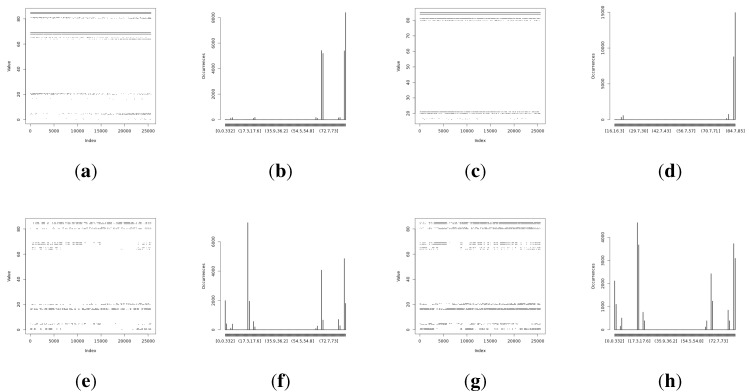
Initial experimental results of the harvested entropy by the fourth method in conjunction with the particular on-board sensor. (**a**) Light (1) sensor entropy; (**b**) distribution from the Light (1) sensor; (**c**) the Light (2) sensor entropy; (**d**) distribution from the Light (2) sensor; (**e**) humidity sensor entropy; (**f**) distribution from the humidity sensor; (**g**) temperature sensor entropy; (**h**) the distribution from the temperature sensor.

The full Shannon entropy and min-entropy comparison of different entropy-harvesting methods is presented in Figure 3. The visual representation and occurrence distributions are presented for Method 1 in Figure 4, Method 2 in Figure 5, Method 3 in Figure 6 and Method 4 in Figure 7.

Initial results have shown that the highest entropy values for every on-board sensor are achieved for the first method. Thus, during the next experiments, only the first method is used.

### 5.2. Long-Term Method Validation

The second experiment is devoted to assess the impact on the amount of entropy during the long-term random value harvesting. For this purpose, for every on-board sensor, over 300,000 random values are gathered during the time span of one month. The home-office environment measurement conditions are upheld. The unmodified version of the first method is used during this experiment; thus, the memory and timing properties have not changed.

**Figure 8:**
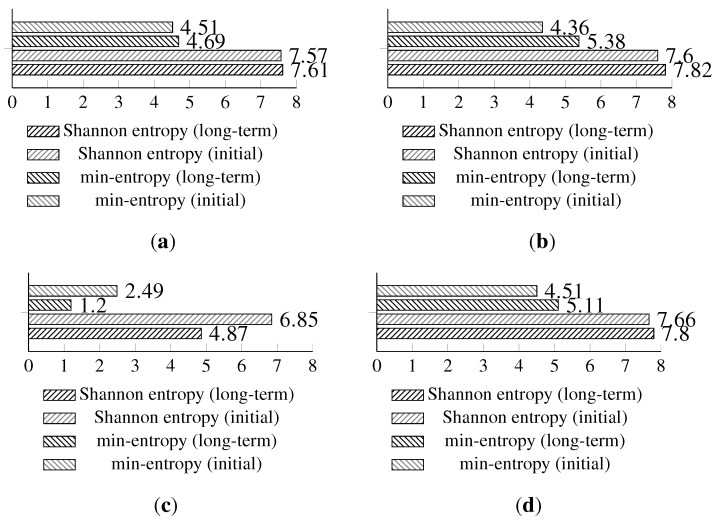
Comparison of Shannon entropy and min-entropy estimations of random bytes of the long-term experiment and the initial results of the analyzed sensors by particular entropy harvesting methods. (**a**) Light (1); (**b**) Light (2); (**c**) humidity; (**d**) temperature.

The long-term entropy harvesting experiment in general has shown minimal variation of the gathered entropy amount. For the Light (1), Light (2) and temperature sensors’ results, entropy estimators showed a small increase. Humidity sensor results are significantly different from the previous experiment with major drops in the entropy values. For the min-entropy estimator, the drop is more than 50% of the previous results. Shannon entropy-estimated values have decreased from 6.85 down to 4.87 bits per byte of data. This suggests that the humidity sensor does not give a high amount of entropy and should be avoided as the source of such. For the full comparison of the estimated entropy results, refer to Figure 8.

**Figure 9:**
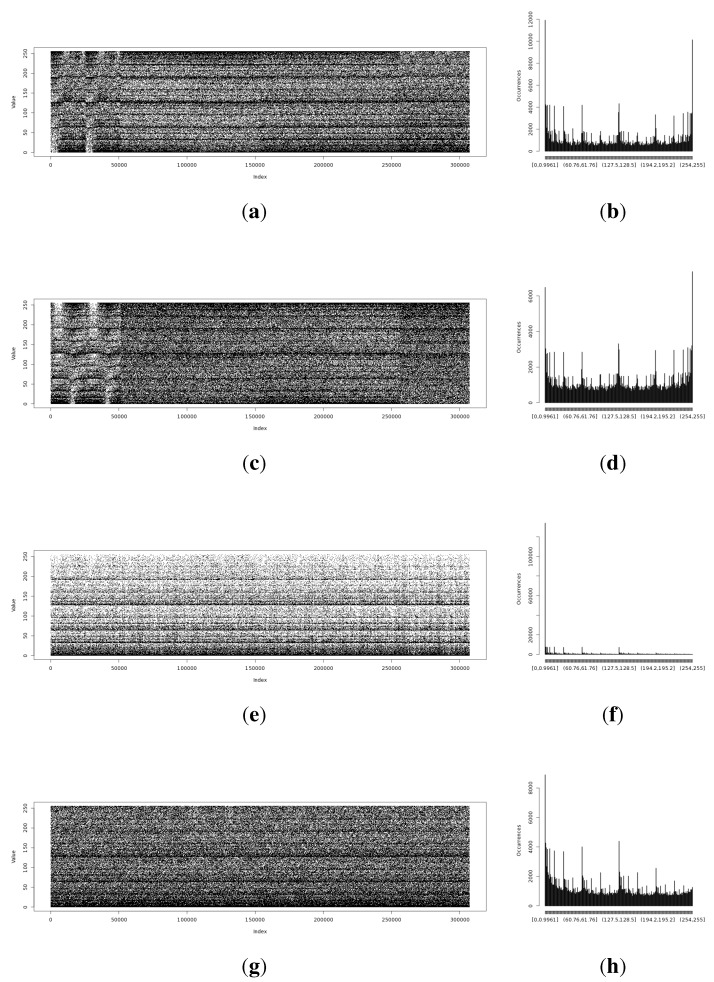
Entropy harvested by the first method from the particular on-board sensor. (**a**) Representation of the harvested entropy collected from the Light (1) sensor; (**b**) occurrence distribution for the Light (1) sensor; (**c**) representation of the harvested entropy collected from the Light (2) sensor; (**d**) occurrence distribution for the Light (2) sensor; (**e**) representation of the harvested entropy collected from the humidity sensor; (**f**) occurrence distribution for the humidity sensor; (**g**) representation of the harvested entropy collected from the temperature sensor; (**h**) occurrence distribution for the temperature sensor.

Random data representations in Figure 9 have given additional information that indicates that the light sensors have resilience issues. Both light sensors have shown pattern variations that can be visually recognized by looking at the occurrence distributions. These artifacts are more significant for the Light (2) sensor. Humidity and temperature sensors have not presented such a visual pattern disturbance. This suggests that light sensors are more prone to be influenced by external conditions than temperature or humidity sensors.

The long-term experiment has shown that the humidity sensor provides the lowest entropy from all of the measured on-board sensors. The min-entropy of the humidity sensor drops by a factor of two during the long-term experiment, so the humidity sensor-based random number generators should be considered as an unreliable source of entropy.

Additionally, it is shown that light sensors are prone to exhibit changes in the patterns of the visual representation of random values. This suggests that light-based sensors are more sensitive to external conditions. Despite that fact, random numbers obtained from light sensors have high entropy values; therefore, they still should be considered as a reasonable source of entropy.

### 5.3. External Impact Evaluation

The isolation experiment setup consisted of two identical devices (alpha, beta) working in the same conditions, concurrently, collecting entropy from the corresponding types of on-board sensors, confined in a sealed, black box. The experiment is designed to test two factors. The first of the tested factors is the resilience of the sensors to the reduction of environmental influence (no external winds, no external light). The second of the tested factors is related to the micro-differences of the electronics of the sensors. The test was designed to show how significant the variations of the measured entropy between the separate identical electronic sensory devices are.

During the experiment, the devices are powered by USB and are not using radio communication. The black box has not been shielded against RF noise, and it is only providing light and temperature protection for the tested on-board sensors. Many scenarios could be designed to improve this evaluation, including high-low temperature or light exposure, RF interference or power source fluctuations analysis, which could give additional information regarding the resilience of the presented approach. As the focus of this research is providing a software means of gathering entropy without introducing hardware changes, the approach employed here is a simple attempt to verify the impact of the light source on the entropy collected from the light sensor or temperature variations for the temperature sensor. The goal has not been to provide a bulletproof solution, but only to evaluate the solution for random number generation for constrained devices.

**Figure 10:**
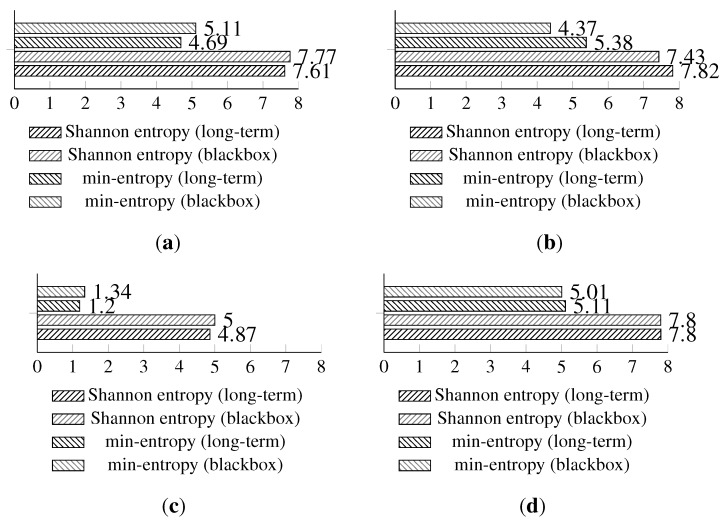
Shannon entropy and min-entropy estimations of the results gathered from the isolation (concatenated results from the alpha and beta devices) and long-term experiments. (**a**) Light (1); (**b**) Light (2); (**c**) humidity; (**d**) temperature.

The isolation experiment has not shown major differences in terms of the Shannon entropy or min-entropy estimations in comparison to the long-term analysis. The full comparison results of the isolation and long-term experiments are presented in Figure 10.

The isolation experiment has shown a discrepancy between the entropy results of alpha and beta devices. Different results are observed for light sensors. The beta sensor is harvesting random values with significantly lower min-entropy. For Light (1), the min-entropy is lowered by 2.39, and for the Light (2) sensor, the drop is estimated as 2.04 bits per byte of data. This result suggests that the amount of the entropy is strongly correlated with in-hardware, electronic variations and noises. The Shannon entropy has not shown any significant variations.

**Figure 11:**
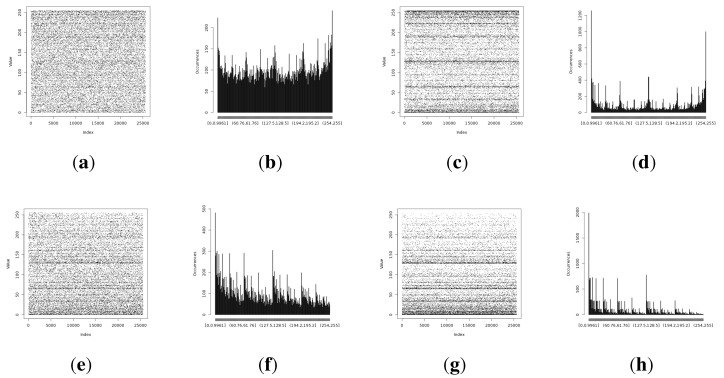

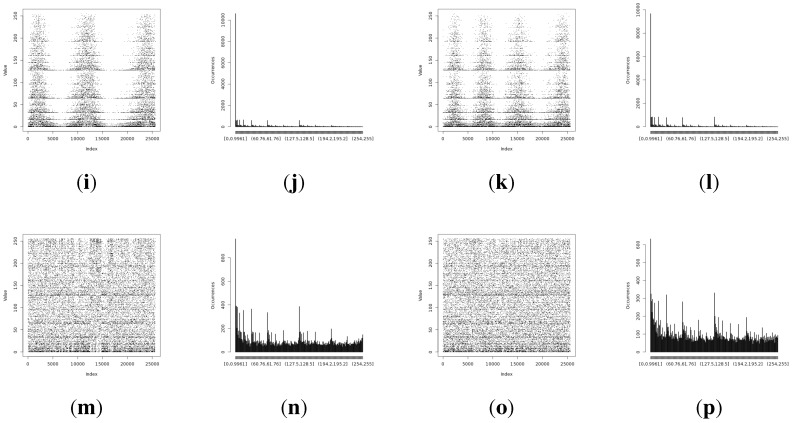
Experiment results representing random values harvested during the isolation experiment. (**a**) Entropy from Light (1) sensor with the original method; (**b**) distribution for the Light (1) sensor with the original method; (**c**) entropy from the Light (1) sensor with the statistically fine-tuned method; (**d**) distribution from the Light (1) sensor with the statistically fine-tuned method; (**e**) entropy from the Light (2) sensor with the original method; (**f**) distribution from the Light (2) sensor with the original method; (**g**) entropy from the Light (2) sensor with the statistically fine-tuned method; (**h**) distribution from the Light (2) sensor with the statistically fine-tuned method; (**i**) entropy from the humidity sensor with the original method; (**j**) distribution from the humidity sensor with the original method; (**k**) entropy from the humidity sensor with the statistically fine-tuned method; (**l**) distribution from the humidity sensor with the statistically fine-tuned method; (**m**) entropy from the temperature sensor with the original method; (**n**) distribution of the harvested entropy collected from the temperature sensor with the original method; (**o**) entropy from the temperature sensor with the statistically fine-tuned method; (**p**) distribution from the temperature sensor with the statistically fine-tuned method.

The temperature and humidity entropy results are similar for both devices. Noteworthy is the fact that the visual representation of the occurrence distribution of the humidity sensor results for alpha and beta (Figure 11i,k) has shown significantly different (more regular) patterns than acquired during previous experiments. This confirms the observation that humidity sensor-based random number generators produce very low entropy and should be avoided.

**Figure 12:**
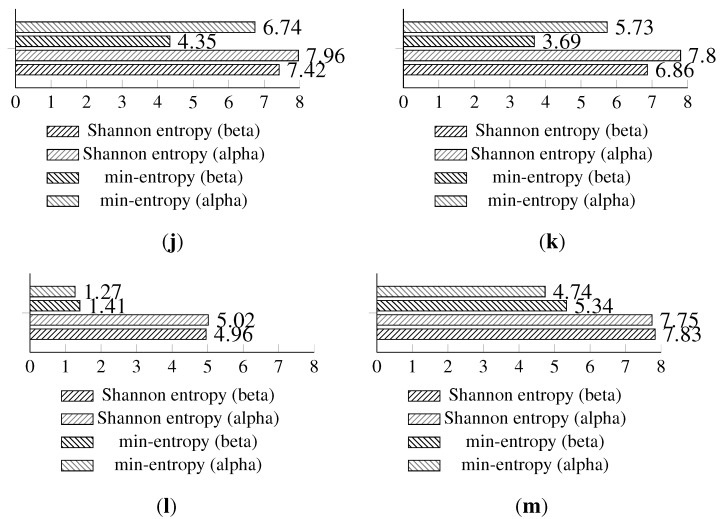
Shannon entropy and min-entropy estimations of the results gathered from the alpha and beta devices during the isolation experiment. (**a**) Light (1); (**b**) Light (2); (**c**) humidity; (**d**) temperature.

A full comparison of the results acquired from the alpha and beta devices during the isolation experiment is presented in Figure 12.

During the isolation experiment, it is shown that light sensors are prone to differ in min-entropy due to the internal hardware micro-differences. The min-entropy variations were significant, but the Shannon entropy was still very high. Therefore, the usage of light-based random number generators should still be a viable option, but caution must be taken.

Additionally, it is confirmed that the humidity-based random number generator creates very low entropy and, in particular, min-entropy. Therefore, humidity-based random number generators should be avoided.

### 5.4. Statistical Fine-Tuning of the Entropy Results

The statistical fine-tuning of the entropy results is the last contribution of this paper. The fine-tuning is applied for the first method and compared to the previous approach. It is tailored for the particular sensor device based on the statistics from the previous results, the historical data of the biased values.

The statistically fine-tuned approach is simple and straightforward. It introduces a table with a subset of eight, up to nine, of the most probable random values with counters with statistics alongside with an additional check that loops back to the entropy harvesting procedure. In terms of functioning, if the value acquired from the entropy-harvesting procedure is one of the most probable ones, then if its counter has not been zeroed, then the random value should be harvested once again. This approach limits the probability of obtaining the most common values for the cost of additional entropy-harvesting time.

In addition to entropy estimations, memory requirements and average harvesting times are measured and compared to the previous approach.

The same assumptions as in the previous experiments are upheld; only one device is used throughout this test.

For the first measurement of the statistically fine-tuned approach, the increase of average harvesting time is calculated. The biggest increase of 94.7% of the runtime is noted for the humidity sensor-based random number generator. Such a result is in line with previous observations, indicating very low entropy for humidity sensor-based methods.

The light-based random number generators’ average running time has increased by 11.8% for Light (1) and 9.6% for Light (2). The lowest increase of the entropy gathering time of 9% is measured for the temperature sensor. This is related to a general observation that the temperature and light sensor-based random number generators produce good entropy.

The second measurement of the statistically fine-tuned method is devoted to estimating the Flash and RAM memory requirements of the implemented solution. All of the statistically fine-tuned implementations have increased the RAM usage up to 23 bytes.

The ROM memory usage has increased for all tested sensors. The light sensor-based random number generators’ Flash memory usage has moved from 488 bytes up to 1756 (259.8%) and 1742 (256.9%) for the Light (1) and Light (2) sensors, respectively. The humidity-based random number generator increased Flash memory requirements from 966 bytes up to 1734 (79.5%). The Flash memory usage for the temperature-based random number generator has increased from 966 up to 1740 bytes (80.1%).

**Figure 13:**
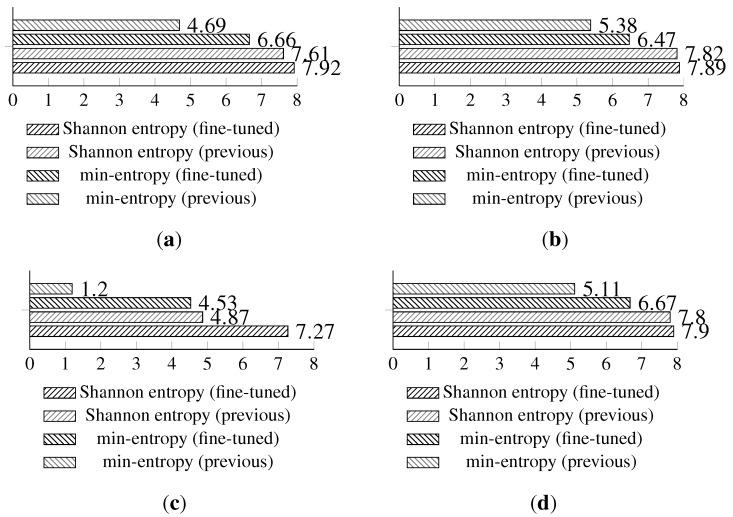
Shannon entropy and min-entropy estimations of previous (long-term results) and statistically fine-tuned versions of the entropy-harvesting method for the particular on-board sensors. (**a**) Light (1); (**b**) Light (2); (**c**) humidity; (**d**) temperature.

The proposed statistical fine-tuning has significantly increased the min-entropy of the gathered random values. The light sensors’ min-entropy has increased by around 42% for Light (1) and 20.2% for Light (2) in comparison to previous results. The temperature sensor min-entropy value is improved by 30.5%. The largest improvement of 277.5% is achieved for the min-entropy of the humidity sensor.

Shannon entropy estimations have increased for all sensors, and they have moved slightly up to around 7.9 bits per byte of data for both temperature and light sensors. The humidity sensor achieved a 49.3% increase for the Shannon entropy.

**Figure 14:**
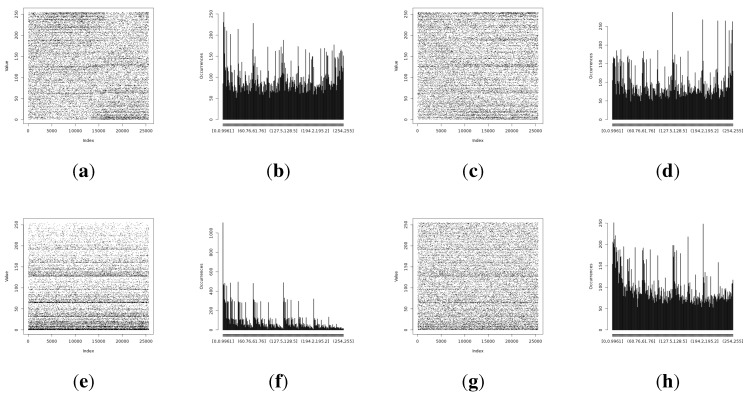
Statistical fine-tuning experiment results for the entropy harvested. (**a**) Light (1) sensor entropy; (**b**) distribution from the Light (1) sensor; (**c**) Light (2) sensor entropy; (**d**) distribution from the Light (2) sensor; (**e**) humidity sensor entropy; (**f**) distribution from the humidity sensor; (**g**) temperature sensor entropy; (**h**) distribution from the temperature sensor.

The full results comparing the basic and statistically fine-tuned methods are presented in Figure 13; additional visual representations with the occurrence distribution are presented in Figure 14.

The proposed statistical fine-tuning has achieved significant improvements for min-entropy estimations of all of the on-board sensor-based random number generators. Shannon entropy results have also slightly improved. The most significant improvements are achieved for humidity sensors at the cost of significantly longer entropy harvesting times.

The most significant flash memory increase of around 257% is noted for light sensor-based random number generators. A more modest increase of around 80% memory is observed for temperature and humidity sensor-based random number generators.

The most beneficial of the statistically fine-tuned sensor-based random number generators are the humidity-based ones. The min-entropy has increased by 277.5% and the Shannon entropy by 49.3%, with the lowest increase of Flash memory usage of 79.5%, but with 94.7% longer average harvesting times. The statistical fine-tuning is the least beneficial for light sensor-based random number generators.

The statistical fine-tuning of the least-significant bit concatenation method of entropy gathering is based on the historical observations of the gathered entropy results. Previous results have shown that particular values are significantly more common than others; this could be managed by applying one of the entropy post-processing mechanisms, like XOR-tree or the von Neumann corrector. The proposed statistical fine-tuning should be regarded only as an example of a simple entropy post-processing mechanism that increases the entropy estimators of the harvested results. Since it is based on historical observations, it should not be reliable for every scenario and could introduce additional bias. Therefore, more observations are required to assess the usability of the statistical fine-tuning, and the analysis of different entropy post-processing mechanisms should be provided.

## 6. Discussion

Primary min-entropy results that were obtained during this research were mostly in parallel with the results presented by the Hennebert, Hossayni and Lauradox in [26]. The measured min-entropy of temperature sensors in [26] is estimated as 3.55 bits per byte of data from the internal sensor and 4.8 bits per byte of data from the external sensor. The on-board temperature sensor that is analyzed during this research has achieved 4.51 bits per byte of data of min-entropy for the standard first method and 6.67 bits per byte of data for the statistically fine-tuned method. That is a much higher entropy value in comparison with the internal temperature sensor results from [26] and also higher for the statistically fine-tuned method in comparison to the external sensors.

The highest Shannon entropy measured in the [26] is for the vibration sensor and is estimated for 7.8 bits per byte of data. The highest measured Shannon entropy of all on-board sensor devices analyzed during this research is observed for the temperature sensor, and it is 7.66 bits per byte of data for the standard method and 7.9 bits per byte of data for the statistically fine-tuned version.

Differences in the values of the entropy between the best-performing methods were not very substantial. Because all of the estimated entropy results have not been presented in [26], the impact of the methods presented in this paper on the entropy is not conclusive. Additional metrics that could be compared with the results from this research have not been presented in [26].

Due to the fact that in [27], the authors are using a very different development environment (*i.e.*, operating system and constrained device processors), the comparisons presented below have more of a symbolic than a conclusive value.

The memory requirements that are presented in this research are significantly lower than the results presented by Francillon and Castelluccia in [27].

ROM memory requirements for the solution presented in [27] are measured as 11,086 bytes for the MICA2 platform and 9832 bytes for the MICAz platform. The highest ROM memory requirement for the solutions analyzed during this research is only 998 bytes for the temperature and humidity-based solution using the most memory consuming method (*i.e.*, the third method). The lowest ROM memory requirement is measured for the first method with the light sensor and is 488 bytes. The solutions presented in this research achieved ROM memory savings of around 90% for humidity/temperature-based methods and of around 95% for light-based methods. For the statistically fine-tuned method, the ROM memory usage increased by around 257% for light-based and 80% for temperature and humidity-based random number generators. The ROM memory usage is a very important factor for very constrained devices and their applications.

RAM memory requirements presented in [27] are measured as 471 bytes for the MICA2 platform and 416 bytes for the MICAz platform. RAM requirements for solutions presented in this research are evaluated for one byte for temperature/humidity sensor-based methods and four bytes for the light sensor-based methods. It should be mentioned that the solutions presented in this research are designed to generate only one byte of the random data in contrast to the solution presented in [27], where it generates eight bytes in one execution. The statistically fine-tuned version requires 23 bytes of RAM for every sensor-based random number generator.

Processing times presented in [27] are estimated at around 146.2 ms (with EEPROM operations) or 4.5 ms (without EEPROM operations), calculated as the sum of random number generation, initialization and entropy accumulation for getting one byte of entropy. On the one hand, the results presented by the authors of [27] are significantly smaller than the time results for temperature/humidity-based methods (1.6 s for Method 1). On the other hand, the processing time results for light sensor-based methods are significantly shorter (1.22 ms for Method 1). The statistically fine-tuned version increased the entropy average harvesting time by 94.7% for humidity sensors and by around 10% for both light sensor and temperature-based random number generators.

The authors of [27] have not measured the amount of entropy of their solution.

## 7. Conclusions

The obtained results are consistent with the assumption that the most entropy could be harvested using a method that concatenates only the least-significant bit of the data read from the sensor device. Least-significant bits of the sensory measurement are prone to various kinds of disorders, the outcome of which is hard to predict. The precision of the measurement, the unpredictability of the external environment and the internal electronic noise are the main factors that contribute to the highest entropy of the sensor-based least significant bit random number generators.

The long-term experiment has shown that the humidity sensor produces the lowest entropy from all of the tested on-board sensors. The min-entropy of the humidity sensor drops by a factor of two; therefore, it should be considered as an unreliable source of entropy. Additionally, it is shown that light sensors are prone to exhibit changes in the pattern of the visual representation of the occurrence of random values. This leads to the conclusion that the light-based sensors are more sensitive to external conditions than other sensors.

The isolation experiment has shown that light sensors are prone to variations in min-entropy due to the internal hardware micro-differences. Min-entropy variations were significant, but the Shannon entropy still was high. In addition, the isolation experiment results have confirmed that the humidity-based random number generator produces very low entropy and, in particular, min-entropy.

The proposed statistical fine-tuning has given significant improvement to min-entropy estimations of all on-board sensor-based random number generators. The Shannon entropy results have also slightly improved. The most significant improvements are achieved for humidity sensors. The most significant Flash memory increase of around 257% is noted for light sensor-based random number generators. A more modest increase of memory is observed for temperature and humidity sensors of around 80%. The statistical fine-tuning method has been the most beneficial for random number generators that have been using humidity sensor as source of the entropy. The min-entropy has increased by 277.5% and the Shannon entropy by 49.3%, with the lowest increase of Flash memory usage of 79.5%, but with 94.7% longer average harvesting times. The statistical fine-tuning is the least beneficial for light sensor-based random number generators.

The best source of entropy has come from the conjunction of the statistically fine-tuned first method and the temperature sensor. The average time required to harvest one byte of entropy is estimated at less than 1.9 s. Such a delay to generate only one bit of entropy is unacceptable, especially for real-time usage in the communication stack.

The most efficient source of entropy with high values of min-entropy and Shannon entropy and exceptionally quick entropy average harvesting times of around 1.3 ms per byte of entropy are light sensors.

Overall, the research results presented in this paper have shown that sensor-based random number generators can be an excellent source of entropy with very minimal RAM and Flash memory requirements. The presented approach could be used as a very good base for future work on true random number generators for the security mechanisms in the constrained environments of the Internet of Things.

The future development of potential sources of entropy for constrained devices will be devoted to analyzing different types of sensory devices in conjunction with different IoT-capable devices. One of the most important sensors that will be analyzed will be Sensirion SHT21, which is part of the Bluetooth Smart development board. The correlation of internal electronic noise influences and dependencies will also be pursued.

An identical methodology will be applied to the analysis of entropy gathered from an analogue microphone. Such a device is also an on-board device of the Bluetooth Smart development board.

The experimental evaluation in the following research will employ the NIST [37] and BSI [38] evaluation methodology for providing more precise and rigorous evaluation of the proposed least-significant bits concatenation method. The application of the mentioned entropy evaluation methodologies requires gathering large numbers of entropy samples, which is strongly limited by the long entropy generation time on the TelosB motes. Enough samples will be collected and an extended evaluation will be provided in the future.
